# Analytical validation of BD‐tau Advantage PLUS kit with clinical corroboration in a traumatic brain injury cohort

**DOI:** 10.1002/alz70856_106909

**Published:** 2026-01-08

**Authors:** Michel N Nafash, Sarah E. Svirsky, Xuemei Zeng, Yijun Chen, Alexandra Gogola, Julia K. Kofler, Dana L Tudorascu, C. Elizabeth Shaaban, Jennifer H Lingler, Tharick A Pascoal, William E Klunk, Victor L. Villemagne, Sarah B Berman, Robert Sweet, Milos D. Ikonomovic, Beth E. Snitz, M. Ilyas Kamboh, Ann D Cohen, Oscar L Lopez, David O Okonkwo, Ava M Puccio, Thomas K Karikari

**Affiliations:** ^1^ University of Pittsburgh, Pittsburgh, PA, USA; ^2^ University of Pittsburgh School of Medicine, Pittsburgh, PA, USA; ^3^ University of Pittsburgh, School of Medicine, Pittsburgh, PA, USA

## Abstract

**Background:**

BD‐tau has emerged as a promising biomarker in predicting Alzheimer's disease‐specific neurodegeneration. However, to our knowledge, there is only one commercially available but not fully validated assay, the BD‐tau Advantage Plus kit (BD‐tau Adv Plus) from Quanterix, for measuring BD‐tau in plasma. This study aims to validate BD‐tau Adv Plus, both analytically and clinically, to evaluate its reliability as a blood‐based BD‐tau assay for clinical research.

**Method:**

Using the Quanterix® Simoa HD‐X analyzer, we evaluated the following analytical metrics: selectivity, recovery, dilution linearity, sample stability, and LLOQ. We also compared BD‐tau levels in plasma versus serum using blood collected from participants (*n* = 48) enrolled in the Pittsburgh Alzheimer's Disease Research Center (Pitt‐ADRC) to determine the matrix effect. Finally, we used a Pittsburgh traumatic brain injury (TBI) cohort with plasma (*n* = 64) and CSF (*n* = 20) samples to evaluate its clinical performance in TBI settings.

**Result:**

Repeated measurements of 3 plasma samples across 18 analytical runs indicated average intra‐plate and inter‐plate CVs at 8.00% and 4.34%, respectively. An average drift of 11.1% (decrease) was observed from the start to the end of a full plate run. Robust linearity was observed between 4 to 16‐fold dilutions. Spiked recovery experiments showed high recoveries when spiked at 140 pg/ml (89.3%) and 75 pg/ml (88.5%) but low when spiked at 9 pg/ml (27.8%). Sample stability tests showed a slight increasing trend with increasing freeze/thaw cycles. BD‐tau Adv Plus showed strong selectivity towards BD‐tau against peripheral‐tau (PNS‐tau), with drastically lower signals in samples spiked with PNS‐tau compared to BD‐tau. Strong positive correlations were observed between plasma and serum in the Pitt‐ADRC cohort (*r* = 0.8392; *p* <0.0001), as well as between plasma and CSF (*n* =  20 pairs, r=0.6150, *p* = 0.0039) in the TBI cohort. Lastly, BD‐tau effectively distinguished severe‐acute TBI, chronic‐mixed TBI, and control groups. In severe TBI patients, significant correlations were observed between BD‐tau and classical AD/TBI biomarkers such as, *p*‐tau217 (plasma: r=0.5761, *p* = 0.0005; CSF: r=0.9667, *p* = 0.0002 in CSF), NfL (plasma: r=0.8910, *p* = 0.0001), and GFAP (plasma: r=0.5424, *p* = 0.0011).

**Conclusion:**

The BD‐tau Advantage PLUS kit produced robust BD‐tau readings that were proven reliable in detecting TBI and distinguishing injury severity.

